# Laterality of Stance during Optic Flow Stimulation in Male and Female Young Adults

**DOI:** 10.1155/2015/542645

**Published:** 2015-10-11

**Authors:** Michela Persiani, Alessandro Piras, Salvatore Squatrito, Milena Raffi

**Affiliations:** Department of Biomedical and Neuromotor Sciences, University of Bologna, 40126 Bologna, Italy

## Abstract

During self-motion, the spatial and temporal properties of the optic flow input directly influence the body sway. Men and women have anatomical and biomechanical differences that influence the postural control during visual stimulation. Given that recent findings suggest a peculiar role of each leg in the postural control of the two genders, we investigated whether the body sway during optic flow perturbances is lateralized and whether anteroposterior and mediolateral components of specific center of pressure (COP) parameters of the right and left legs differ, reexamining a previous experiment (Raffi et al. (2014)) performed with two, side-by-side, force plates. Experiments were performed on 24 right-handed and right-footed young subjects. We analyzed five measures related to the COP of each foot and global data: anteroposterior and mediolateral range of oscillation, anteroposterior and mediolateral COP velocity, and sway area. Results showed that men consistently had larger COP parameters than women. The values of the COP parameters were correlated between the two feet only in the mediolateral axis of women. These findings suggest that optic flow stimulation causes asymmetry in postural balance and different lateralization of postural controls in men and women.

## 1. Introduction

The human upright stance is characterized by continuous movements of the body similar to an inverted pendulum [1]. Vision provides the nervous system with information regarding the position and movements of elements present in the environment relatively to the body, playing an important role in the postural stabilization. The characteristic pattern of visual stimuli that provides information of self-motion and the environmental structure is defined as “optic flow” [2, 3]. The optic flow originates from the focus of expansion (FOE), a point of the visual scene that corresponds to the final destination of self-motion. The neural mechanisms integrate visual, vestibular, and proprioceptive inputs of self-motion perception to generate the typical body oscillation defined as body sway. The body sway is regulated by the neuromotor system and is considered a consequence of small postural oscillations. These small postural oscillations reflect the regulatory activity of the several control loops of stabilization of an unstable structure, such as the human body, for maintenance of balance [4, 5].

Until now, several studies have focused on the maintenance of balance control, looking at the variation of the center of pressure (COP) trajectory. The COP analysis with bilateral force plate can be useful for assessing postural behavior related to each foot in healthy individuals [6, 7]. Few studies have addressed the laterality or asymmetry during quiet stance; however, these studies were performed with the eyes open or closed or under two-dimensional visual stimulation [8–10].

In a previous paper, we showed that foveal, peripheral, and full field optic flow stimulations evoke different muscular activations in the right and left leg and different directions of oscillation in men and women [11]. Thus, the aim of this paper was to verify whether the different oscillations caused by foveal, peripheral, and full field optic flows depend on the variations of specific COP parameters in each leg. Results showed that optic flow significantly affected the COP parameters, and each foot had a specific contribution to postural control that was not evident in the global data.

## 2. Methods

For this study, we reexamined the data of the experiments performed on 24 right-handed and right-footed subjects [11], 12 women and 12 men, ranging from 20 to 30 years (average age was 24.5). Average height and weight plus standard deviation (SD) was 167 ± 5 cm and 62 ± 5 kg for women and 178 ± 6 cm and 72 ± 5 kg for men. All subjects voluntarily participated in the experiments. The experimental protocol was approved by the Institutional Ethic Committee of the University of Bologna. Recordings were performed in accordance with the ethical standards laid down in the 1964 Declaration of Helsinki. The subjects included in the sample practiced a moderate physical activity (i.e., no more than 1 hour three times a week). None of the subjects had any history of gait or posture disorders or injuries in the previous two years; all of them had normal or corrected-to-normal vision.

### 2.1. Experimental Procedure and Stimuli

The experimental paradigm and visual stimuli are identical to those described in a previous publication [11]. The experiments were performed in a dark room. Optic flow stimuli and fixation point were presented by a retro video projector (Sony VPL EX3) positioned 415 cm away from a translucent screen that covered 135 × 107° of visual field, placed 115 cm from the subjects' eyes.

**Figure 1:**
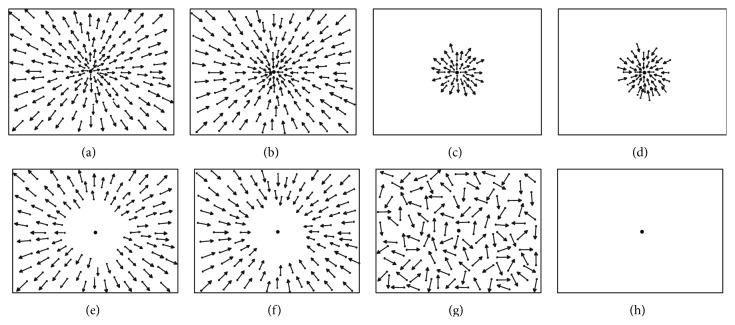
Optic flow stimuli. Arrows represent the velocity vectors of moving dots. (a) Full field expansion. (b) Full field contraction. (c) Foveal expansion. (d) Foveal contraction. For the foveal stimuli, the stimulated area had a radius of 7°. (e) Peripheral expansion. (f) Peripheral contraction. For the peripheral stimuli, the blank area in the center had a radius of 20°. (g) Random motion stimulus. (h) Baseline (fixation in the dark).

Optic flow stimuli consisted of white dots (1.3 cd/m^2^) of 0.4° size, which moved at a speed of 5°/s. The stimuli were expanding and contracting flows originating from a central FOE. The fixation point consisted of a white dot of 0.6° always positioned in the middle of the screen. The focus of expansion was always in the center of the screen. Expansion and contraction optic flows were presented as full field (Exp and Contr, resp., Figures 1(a) and 1(b)), foveal (ExpF and ContrF, resp., Figures 1(c) and 1(d)), or peripheral (ExpP and ContrP, resp., Figures 1(e) and 1(f)) stimuli. A random dot motion stimulus was used as a control (random, Figure 1(g)). For baseline trials, stabilometric activity was recorded while the subject fixated on the fixation point without visual stimulation (Figure 1(h)). Stimuli were created using MATLAB Psychophysical Toolbox (The MathWorks Inc.) and had the same dot density with respect to the retinal stimulated area. Full field stimulus is 1155 dots. Foveal stimulus is 36 dots. Peripheral stimulus is 992 dots.

The stabilometric data were acquired using two Kistler force platforms (number 9286BA). Subjects were instructed to place a foot on each platform before the beginning of each trial. The platforms were marked to normalize posture and to control the subject's distance from the screen. Subjects had to look at a white fixation point (0.6°), which was also the FOE of the optic flow stimuli, always positioned in the center of the screen and adjusted to the height of each subject. The stimulus was present during the entire trial duration and trial onset was determined by the stimulus onset.

### 2.2. Data Analysis

We acquired 5 trials for each stimulus condition and 4 trials at baseline (i.e., fixation in the dark without visual stimulation, Figure 1(h)). Each stimulus lasted about 30–35 s. Stabilometric signals were recorded at 1000 Hz, then low-pass filtered at 15 Hz, and resampled at 250 Hz. We recorded ground reaction forces and COP measures from each foot by the two platforms. We analyzed both anteroposterior COP and mediolateral COP of each foot using SMART Analyzer (BTS Bioengineering Inc.) and MATLAB (The MathWorks, Inc.). Subsequently, we computed the global COP according to the following formula [12]:

(1) $$\begin{array}{c} {\text{COP}}_{\text{global}}=\frac{{\text{COP}}_{\text{L}}*R_{\text{VL}}}{\left(R_{\text{VL}}+R_{\text{VR}}\right)}+\frac{{\text{COP}}_{\text{R}}*R_{\text{VR}}}{\left(R_{\text{VL}}+R_{\text{VR}}\right)}, \end{array}$$

where *R*_VL_ and *R*_VR_ are the vertical reaction forces from left and right feet, respectively. The analysis was performed in the first 25 s of each trial.

In this study, we computed five measures referring to the COP of each foot and COP_global_: (1) the anteroposterior range of oscillation (APO), which is the difference between the maximum and minimum range of oscillation in anteroposterior direction [13], (2) the mediolateral range of oscillation (MLO), which is the difference between the maximum and minimum range of oscillation in the mediolateral direction [13], (3) the anteroposterior COP velocity (VelAP), (4) the mediolateral COP velocity (VelML), the two latter measurements reflecting the total distance travelled by the COP over time on each axis [13–16], and (5) the COP area (Area), quantified within the 95% confidence ellipse, which is the enclosed area covered by the COP as it oscillates within the base of support [17].

We first computed the percentage of loading in the right and left foot using Smart-Analyzer software (BTS Bioengineering Inc.) and MATLAB (The MathWorks Inc.). The values of the percentage of loading were then analyzed with a multivariate ANOVA (within-subject factor: stimuli; between-subject factors: side and gender).

Then, we analyzed the COP parameters APO, MLO, VelAP, VelML, and Area using Sway and Smart-Analyzer software (BTS Bioengineering Inc.) and MATLAB (The MathWorks Inc.). The analysis was performed separately for measurements of each limb and global. To analyze the influence of optic flow stimuli on postural control, we performed a repeated-measure ANOVA in which optic flow stimuli and side (right, left, and global) were the within-subject factors, while gender was the between-subjects factor.

After having assessed the effects of stimuli, side, and gender, we then analyzed in depth the relationship between the left and right feet in response to visual stimuli using a bivariate Pearson linear correlation analysis.

Lastly, we looked at the degree of variation of the right and left foot in the five COP parameters using the coefficient of variation (CV) computed as the ratio of the standard deviation to the mean. The CV was computed for each trial of each stimulus in each subject. Then, values for all subjects in each condition and group were averaged.

## 3. Results 

### 3.1. Limb Loading

**Figure 2:**
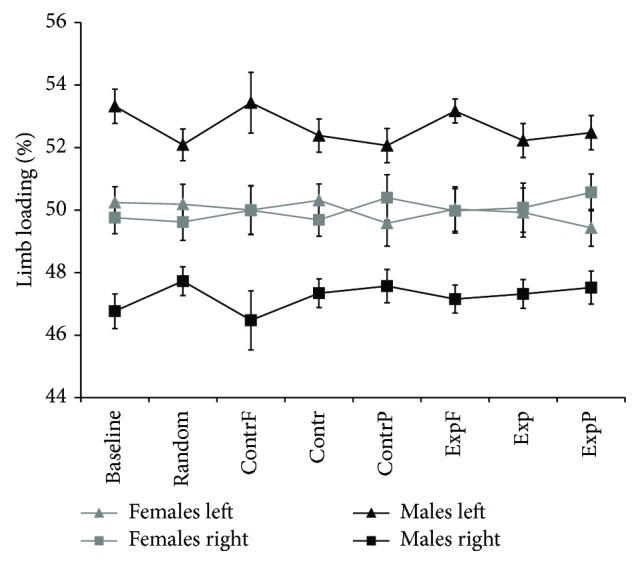
Average values of left and right percentage of loading in the right and left foot of men and women. Data are shown for all stimuli and baseline. Each data point shows mean ± standard error (SE). ContrF: foveal contraction, Contr: full field contraction, ContrP: peripheral contraction, ExpF: foveal expansion, Exp: full field expansion, and ExpP: peripheral expansion.

To quantify the asymmetry, we first computed the limb loading. Mean values of the percentage of loading are shown in Figure 2. Women had an almost equal load, while men consistently loaded the left leg more than the right. The results of the multivariate ANOVA (see Methods) showed an effect of side in all stimuli (*F*(8,35) = 10,57, *p* < 0.001) and an interaction effect of side × gender in all stimuli (*F*(8,35) = 7,74, *p* < 0.001). No main effect of gender was found (*F*(8,35) = 0.31, *p* = 0.95).

### 3.2. Effect of Stimuli, Side, and Gender on Postural Responses

All COP parameters showed significant main effects of stimuli, side, and gender as summarized in Table 1. VelML did not show a significant main effect of gender but showed significant interaction effects (stimulus × gender and stimulus × gender × side). Area showed an interaction effect between stimulus and side.

**Figure 3:**
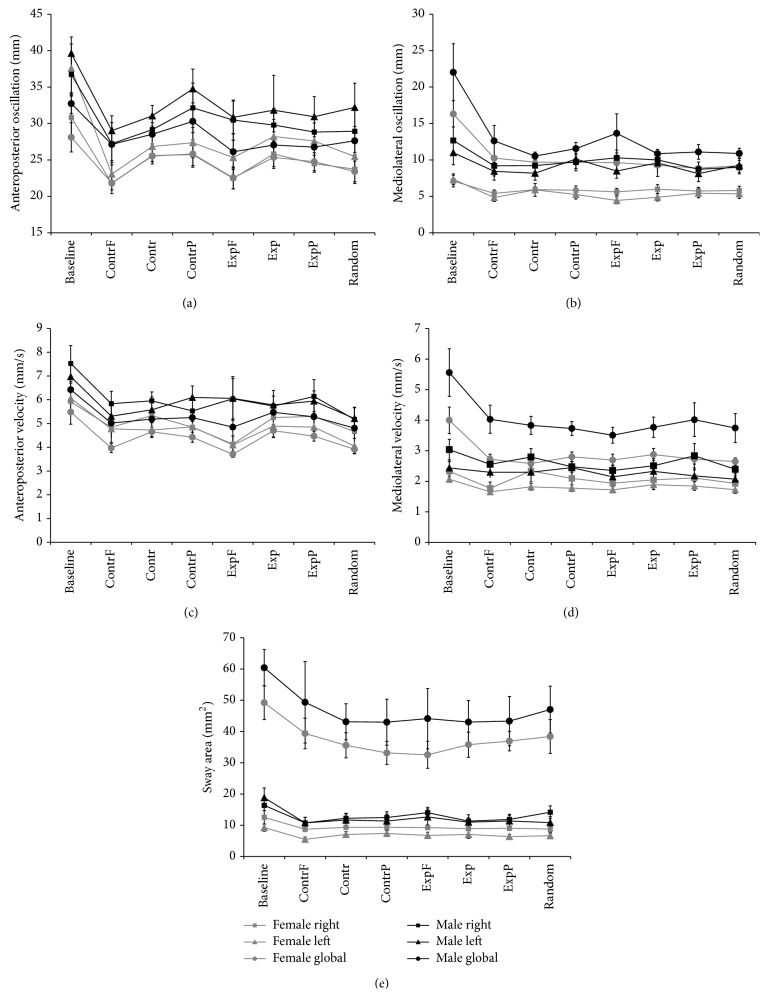
Average values of COP parameters in the left and right limb and global data. Values are shown for men and women during optic flow stimuli and baseline. (a) Anteroposterior range of oscillation (APO). (b) Mediolateral range of oscillation (MLO). (c) Anteroposterior velocity (VelAP). (d) Mediolateral velocity (VelML). (e) Sway area (Area). Each data point shows mean ± standard error (SE). Conventions are as in Figure 2.

The results of the between-subjects analysis (ANOVA, see Methods) showed that, among the COP parameters, MLO showed more differences between men and women. The gender effect was examined in each stimulus of the right and left leg allowing the analysis in 16 conditions. A significant effect was found in almost all stimuli (14/16). The two nonsignificant effects were found in expansion (*p* = 0.14) and foveal contraction (*p* = 0.08) of the left foot. No difference emerged in the stimuli of the MLO_global_. In APO, however, significant gender effects were found for foveal (*p* < 0.024) and peripheral contraction (*p* < 0.028) stimuli in the left leg, while no differences were found in the APO_global_. The VelAP and VelML showed similar results: in VelAP, a significant gender effect was found in the left foot only for baseline, random, and foveal stimuli (*p* < 0.05), while in VelML significant gender differences were observed in the left foot for baseline, random, and peripheral contraction stimuli (*p* < 0.05). Similar to MLO, the Area parameter showed a significant gender effect in the right and left foot in 13 out of 16 stimuli (ANOVA, *p* < 0.05). The three nonsignificant effects were found for expansion (*p* = 0.09) and peripheral contraction (*p* = 0.08) of the left foot and foveal contraction of the right foot (*p* = 0.22). No differences were found for the Area_global_ parameter.  Figure 3 shows the mean values of the COP parameters in both feet and the global data for men and women. All parameters yielded larger values in men. The left foot had larger values of APO and Area (Figures 3(a) and 3(e)), while the right foot showed higher values in MLO, VelAP, and VelML (Figures 3(b)–3(d)).

### 3.3. Correlation Analysis

**Figure 4:**
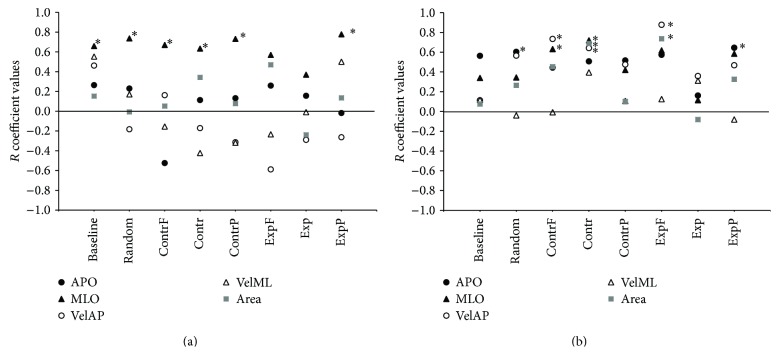
Correlation coefficients for the correlation analysis between the right and left foot. (a) Women. (b) Men. Asterisks indicate significant values (bivariate Pearson correlation, *p* < 0.05). Conventions are as in Figures 2 and 3.

A bivariate Pearson correlation was used to test whether the relationship between the right and left foot in each COP parameter was linear. The analysis was performed separately for men and women on left versus right foot for all stimuli and baseline values of each COP parameter. In women (Figure 4(a)), significant linear correlations between the two feet were found only in MLO (baseline: *R*(9) = 0.659, *p* = 0.05; random: *R*(11) = 0.737, *p* = 0.01; foveal contraction: *R*(11) = 0.67, *p* = 0.02; contraction: *R*(11) = 0.634, *p* = 0.036; peripheral contraction: *R*(11) = 0.731, *p* = 0.011; peripheral expansion: *R*(12) = 0.778, *p* = 0.003). The values of the right and left foot in the COP other parameters showed very low correlation coefficients, often negative (Figure 4(a)). Men, however, showed few significant correlations between right and left foot COP values (Figure 4(b)) but the two feet seem to have more similar movements than those of women (APO random: *R*(11) = 0.603, *p* = 0.049; APO peripheral expansion: *R*(11) = 0.644, *p* = 0.032; VelAP foveal contraction: *R*(11) = 0.733, *p* = 0.01; VelAP contraction: *R*(11) = 0.641, *p* = 0.033; VelAP foveal expansion: *R*(12) = 0.877, *p* < 0.001; MLO foveal contraction: *R*(10) = 0.631, *p* = 0.05; MLO contraction: *R*(9) = 0.72, *p* = 0.029; Area contraction: *R*(11) = 0.688, *p* = 0.019; Area foveal expansion: *R*(11) = 0.736, *p* = 0.01).

### 3.4. Variation in the COP Parameters

**Figure 5:**
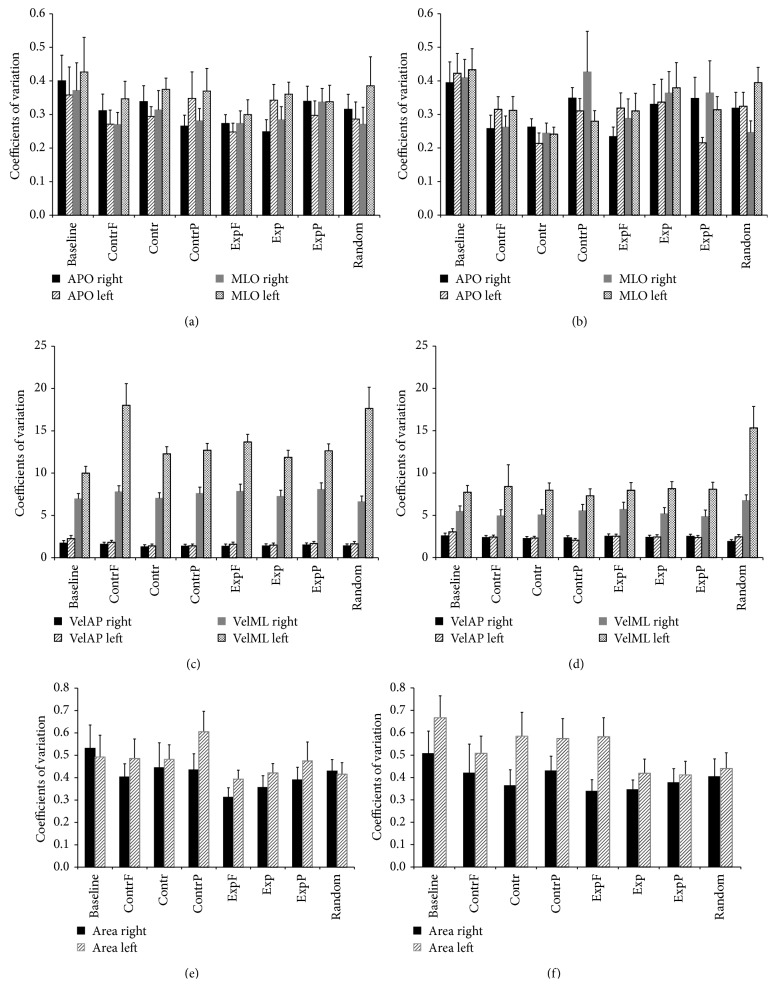
Coefficients of variations of COP parameters across the right and left feet in men and women. (a) Women anteroposterior range of oscillation (APO) and mediolateral range of oscillation (MLO). (b) Male APO and MLO. (c) Women anteroposterior velocity (VelAP) and mediolateral velocity (VelML). (d) Men VelAP and VelML. (e) Female sway Area. (f) Male sway Area.

To examine the variability of postural adjustments during optic flow stimulation, we computed the CV for the five COP parameters in the right and left foot. MLO consistently showed greater variability than APO. Baseline stimuli always had the highest CV, indicating that the absence of visual stimulation caused a greater instability. In women, different variability was observed in the left and right foot: MLO_left_ always showed higher CV than MLO_right_, while, in almost all stimuli, APO_right_ showed higher CV than APO_left_ (Figure 5(a)). In men, MLO had still higher variations than APO; however, they were smaller when compared to those of women (Figure 5(b)). The greatest variations were observed in the COP velocity (Figures 5(c) and 5(d)). In both men and women, VelML always showed greater variations than VelAP, suggesting that subjects consistently experienced a loss of balance control on the mediolateral axis. Both genders showed greater variability for Area of the left foot for the majority of stimuli (Figures 5(e) and 5(f)). Men showed the greatest variability of the COP Area.

As these observations on the CV were largely descriptive, the CV values were further analyzed to quantify the variability related to gender and foot. A one-way ANOVA, with side as between-subject factor and stimuli as within-subject factor, was performed separately for men and women. Significant differences between the left and right feet were found only in women in VelML for all visual stimuli (foveal contraction: *F*(1,23) = 4.69, MS = 630.29, *p* = 0.041; contraction: *F*(1,23) = 20.73, MS = 166.48, *p* < 0.001; peripheral contraction: *F*(1,21) = 15.23, MS = 144.67, *p* = 0.001; foveal expansion: *F*(1,21) = 24.05, MS = 187.27, *p* < 0.001; expansion: *F*(1,23) = 13.61, MS = 125.18, *p* = 0.001; peripheral expansion: *F*(1,23) = 12.7, MS = 125.26, *p* = 0.002; random: *F*(1,22) = 5.04, MS = 696.75, *p* = 0.036; baseline: *F*(1,23) = 3.84, MS = 55.72, *p* = 0.063). No significant differences between the two feet were found in the other parameters in men.

## 4. Discussion

The optic flow is a key input for maintaining postural stability during self-motion [18]. The human body is fundamentally asymmetrical, manifesting in the functional anteroposterior and mediolateral asymmetries observed in balance control [19]. The aim of this study was to investigate whether the body sway during foveal, peripheral, or full field optic flow stimulation is lateralized and whether anteroposterior and mediolateral components of specific COP parameters of the right and left foot differ between the two genders.

### 4.1. Limb Load Asymmetry

An important issue in studying postural asymmetry is limb loading. Some evidence seems to support the idea that healthy subjects unequally distribute their weight across the two feet in conditions of eyes open and closed [9, 20]. Our female subjects showed an almost even limb loading while men loaded the left limb more than the right. This is a first indication of gender differences in the postural control during optic flow stimulation. The left loading preference of men, irrespective of handedness and footedness, can be explained by the different muscular activity. Indeed, the electromyography recordings performed in the previous study showed that men showed the greatest activation in the left thigh muscles [11]. This is also supported by the fact that male soccer players have a better standing balance on the nondominant leg, probably as a consequence of many hours of soccer practice during which they maintain standing balance for a few seconds on the nondominant leg for kicking the ball with the dominant foot to have more precision [21]. Practicing physical activity seems to enhance the inter-leg differences, because it has been shown that dominance does not interfere in the evaluation of single-foot balance among healthy sedentary individuals [22].

### 4.2. Contribution of Individual Leg on Postural Control

Footedness entails postural asymmetry [23]. All subjects were right-footed [11]. This, together with our analysis model, allowed us to broaden the knowledge on the contribution of each leg to postural control during the view of optic flow stimuli. Some authors suggest differential effects on the recurrent dynamics of the individual leg COPs and COP_global_ trajectories [24, 25]. The detailed analysis of left, right, and global data shows that each leg contributes individually to side-by-side postural control, which is not obvious when analyzing the global data. As pointed out by King and coworkers [24, 25], the degree of asymmetry between left leg and right leg COP dynamics differed across all postural stances and COP_global_ dynamics. Analyzing each foot separately revealed variation of postural control in terms of different variability between the left and right foot parameters. The present study emphasizes asymmetries between the two feet in the postural maintenance showing different dynamics between the two feet in each parameter.

### 4.3. Gender Asymmetry

The present results suggest that optic flow stimuli produced different COP oscillations, velocities, and area dimensions. These results point out important characteristics of the feet asymmetry; the fact that the two feet exhibit different values in distinct parameters may indicate that each foot has its own role in balance control. As suggested by Anker and coworkers [14], the muscles of the unloaded leg lose their capacity to generate effective stabilizing ankle torques, while the velocity of COP under the loaded leg increases, reflecting the generation of compensatory ankle moments. Our subjects showed no significant relationship between limb dominance and the side of load preference meaning a continuous load/unload balance between the two feet. The lack of correlations between the behavior of the two feet in the majority of the stimuli and parameters is another indication of gender and limb differences, further emphasized by the different variability observed in the mediolateral velocity of left and right foot in women. These findings seem to suggest that foot asymmetry induces inter-leg coordination dynamics based on postural demands during optic flow stimulation and during increasing difficulty to maintain correct body balance. This might reveal the use of multiple timescale processes within each leg to produce a stable and flexible postural strategy.

Gender differences in brain asymmetry are well documented and may explain the different postural strategies exhibited by men and women. The brain of adult women is, from the functional point of view, less asymmetrical than that of men [26, 27]. A recent study showed a larger left > right asymmetry in women in anterior brain regions and a larger right > left asymmetry in men orbitofrontal, inferior parietal, and inferior occipital cortices [28]. The brain asymmetry is also evident in motor function, as it is known that the gray matter density in the corticospinal tract shows a hemispheric asymmetry related to hand preference, and the maturation of the corticospinal tract during adolescence differs between men and women due to the influence of testosterone [29]. It seems that the leftward asymmetry of the corticospinal tract may reflect an early established asymmetry in the corticomotoneuronal fibres. The present results, together with those of previous findings [30], suggest that men and women adapt differently to cortical and corticospinal asymmetry leading to different behaviors of the right and left limb.

## 5. Conclusions

This study provides new evidence on the postural strategy used by men and women in the control of stance under visual optic flow stimulation. The feet asymmetry observed during optic flow stimulation causes specific inter-leg coordination dynamics necessary to maintain the control of posture. This might suggest that the postural control system uses various mechanisms within each leg to produce the most appropriate postural response to interact with the extrapersonal environment.

## Figures and Tables

**Table 1:** Full statistical information for the repeated-measure ANOVA in which optic flow stimuli and side (right, left, and global) were the within-subject factors, while gender was the between-subjects factor.

	APO	MLO	VelAP	VelML	Area
Side	*F*(2; 42) = 7.70; MSE = 148.28; **p** = 0.009^**∗**^	*F*(2; 42) = 15.05; MSE = 56.24; **p** = 0.002^**∗**^	*F*(2; 42) = 5.38; MSE = 3.72; **p** = 0.015^**∗**^	*F*(2; 42) = 5.08; MSE = 3.35; **p** = 0.05^**∗**^	*F*(2; 42) = 123.77; MSE = 883.66; **p** < 0.001^**∗**^

Sex	*F*(1; 21) = 5.96; MSE = 489.87; **p** = 0.024^**∗**^	*F*(1; 21) = 14.63; MSE = 151.1; **p** = 0.009^**∗**^	*F*(1; 21) = 5.68; MSE = 27.13; **p** = 0.041^**∗**^	*F*(1; 21) = 6.94; MSE = 4.86; *p* = 0.07	*F*(1; 21) = 4.22; MSE = 1165.7; **p** = 0.05^**∗**^

Stimulus	*F*(7; 147) = 11.73; MSE = 76.48; **p** < 0.001^**∗**^	*F*(7; 147) = 5.43; MSE = 103.56; **p** = 0.035^**∗**^	*F*(7; 147) = 6.49; MSE = 2.53; **p** = 0.002^**∗**^	*F*(7; 147) = 5.72; MSE = 0.22; **p** = 0.001^**∗**^	*F*(7; 147) = 5.30; MSE = 193.62; **p** = 0.001^**∗**^

Stimulus × sex	*F*(7; 147) = 0.55; MSE = 76.48; *p* = 0.706	*F*(7; 147) = 1.54; MSE = 103.56; *p* = 0.259	*F*(7; 147) = 1.34; MSE = 2.53; *p* = 0.281	*F*(7; 147) = 2.71; MSE = 0.22; **p** = 0.036^**∗**^	*F*(7; 147) = 0.25; MSE = 193.62; *p* = 0.891

Side × sex	*F*(2; 42) = 0.45; MSE = 148.28; *p* = 0.524	*F*(2; 42) = 0.31; MSE = 56.24; *p* = 0.67	*F*(2; 42) = 1.16; MSE = 7.2; *p* = 0.335	*F*(2; 42) = 0.38; MSE = 4.75; *p* = 0.635	*F*(2; 42) = 0.8; MSE = 709.51; *p* = 0.393

Stimulus × side	*F*(14; 294) = 1.57; MSE = 41.95; *p* = 0.171	*F*(14; 294) = 1.92; MSE = 130.85; *p* = 0.2	*F*(14; 294) = 0.98; MSE = 0.7; *p* = 0.417	*F*(14; 294) = 1.67; MSE = 1.97; *p* = 0.26	*F*(14; 294) = 2.08; MSE = 72.47; **p** = 0.012^**∗**^

Side × stimulus × sex	*F*(14; 294) = 0.5; MSE = 41.95; *p* = 0.781	*F*(14; 294) = 0.55; MSE = 130.85; *p* = 0.556	*F*(14; 294) = 1.31; MSE = 0.7; *p* = 0.289	*F*(14; 294) = 2.17; MSE = 0.3; **p** = 0.027^**∗**^	*F*(14; 294) = 0.13; MSE = 229.03; *p* = 0.977

Significant values are in bold and marked with an asterisk.

## References

[1] Johansson R., Magnusson M., Akesson M. (1988). Identification of human postural dynamics. *IEEE Transactions on Biomedical Engineering*.

[2] Gibson J. J. (1950). *The Perception of the Visual World*.

[3] Gibson J. J. (1954). The visual perception of objective motion and subjective movement. *Psychological Review*.

[4] Collins J. J., de Luca C. J. (1993). Open-loop and closed-loop control of posture: a random-walk analysis of center-of-pressure trajectories. *Experimental Brain Research*.

[5] Gatev P., Thomas S., Kepple T., Hallett M. (1999). Feedforward ankle strategy of balance during quiet stance in adults. *Journal of Physiology*.

[6] Benvenuti F. (2001). Physiology of human balance. *Advances in Neurology*.

[7] Mizrahi J., Susak Z. (1989). Bi-lateral reactive force patterns in postural sway activity of normal subjects. *Biological Cybernetics*.

[8] Kinsella-Shaw J. M., Harrison S. J., Carello C., Turvey M. T. (2013). Laterality of quiet standing in old and young. *Experimental Brain Research*.

[9] Blaszczyk J. W., Prince F., Raiche M., Hébert R. (2000). Effect of ageing and vision on limb load asymmetry during quiet stance. *Journal of Biomechanics*.

[10] Jonsson E., Seiger A., Hirschfeld H. (2005). Postural steadiness and weight distribution during tandem stance in healthy young and elderly adults. *Clinical Biomechanics*.

[11] Raffi M., Piras A., Persiani M., Squatrito S. (2014). Importance of optic flow for postural stability of male and female young adults. *European Journal of Applied Physiology*.

[12] Winter D. A., Patla A. E., Ishac M., Gage W. H. (2003). Motor mechanisms of balance during quiet standing. *Journal of Electromyography and Kinesiology*.

[13] Winter D. A. (1995). Human balance and posture control during standing and walking. *Gait & Posture*.

[14] Anker L. C., Weerdesteyn V., van Nes I. J. W., Nienhuis B., Straatman H., Geurts A. C. H. (2008). The relation between postural stability and weight distribution in healthy subjects. *Gait and Posture*.

[15] Palmieri R. M., Ingersoll C. D., Stone M. B., Krause B. A. (2002). Center-of-pressure parameters used in the assessment of postural control. *Journal of Sport Rehabilitation*.

[16] Wang Z., Newell K. M. (2012). Asymmetry of foot position and weight distribution channels the inter-leg coordination dynamics of standing. *Experimental Brain Research*.

[17] Kim G. T., Ferdjallah M., Harris G. F. (2009). Fast computational analysis of sway area using center of pressure data in normal children and children with cerebral palsy. *American Journal of Biomedical Sciences*.

[18] Lee D. N., Lishman J. R. (1975). Visual proprioceptive control of stance. *Journal of Human Movement Studies*.

[19] Winter D. A., Prince F., Frank J. S., Powell C., Zabjek K. F. (1996). Unified theory regarding A/P and M/L balance in quiet stance. *Journal of Neurophysiology*.

[20] Gutnik B., Leaver J., Standen C., Longley C. (2008). Inferred influence of human lateral profile on limb load asymmetry during a quiet standing balance test. *Acta Medica Okayama*.

[21] Barone R., Macaluso F., Traina M., Leonardi V., Farina F., Di Felice V. (2011). Soccer players have a better standing balance in nondominant one-legged stance. *Open Access Journal of Sports Medicine*.

[22] Alonso A. C., Brech G. C., Bourquin A. M., Greve J. M. D. (2011). The influence of lower-limb dominance on postural balance. *Sao Paulo Medical Journal*.

[23] Day L. B., MacNeilage P. F. (1996). Postural asymmetries and language lateralization in humans (*Homo sapiens*). *Journal of Comparative Psychology*.

[24] King A. C., Wang Z., Newell K. M. (2012). Asymmetry of recurrent dynamics as a function of postural stance. *Experimental Brain Research*.

[25] Kirby R. L., Price N. A., MacLeod D. A. (1987). The influence of foot position on standing balance. *Journal of Biomechanics*.

[26] McGlone J. (1978). Sex differences in functional brain asymmetry. *Cortex*.

[27] Inglis J., Lawson J. S. (1981). Sex differences in the effects of unilateral brain damage on intelligence. *Science*.

[28] Plessen K. J., Hugdahl K., Bansal R., Hao X., Peterson B. S. (2014). Sex, age, and cognitive correlates of asymmetries in thickness of the cortical mantle across the life span. *The Journal of Neuroscience*.

[29] Hervé P.-Y., Leonard G., Perron M. (2009). Handedness, motor skills and maturation of the corticospinal tract in the adolescent brain. *Human Brain Mapping*.

[30] Haddad J. M., Rietdyk S., Ryu J. H. (2011). Postural asymmetries in response to holding evenly and unevenly distributed loads during self-selected stance. *Journal of Motor Behavior*.

